# What factors contribute to the self-reported oral health status of Arab adolescents? An assessment using a validated Arabic-WHO tool for child oral health (A-OHAT)

**DOI:** 10.1186/s12903-020-1018-8

**Published:** 2020-01-28

**Authors:** Ahmed M. Bokhari, Mir Faeq Ali Quadri

**Affiliations:** Division of Dental Public Health, Department of Preventive Dental Sciences, College of Dentistry, Jazan University, Gizan, 45142 Saudi Arabia

**Keywords:** Validation, Oral health, Assessment tool, Arabic, Child, Oral diseases, Risk factors

## Abstract

**Background:**

The current study was performed; to validate the Arabic version of WHO child oral health assessment tool (A-OHAT), to assess the oral health status of Arab school children and finally to identify the important risk factors associated with the poor teeth and gum conditions of school children.

**Methods:**

A cross-sectional study with two-staged simple random sampling technique was implemented. A-OHAT, a self-assessment tool was subjected to psychometric analyses with the respondents being high school children. The Cronbach’s alpha and the Intra class correlation values were computed. Paired t-test was performed to identify the differences between the readings after repeated administration, followed by the analysis for convergent validity. This tested Arabic-WHO Child-OHAT was administered to collect the data. Univariate, bivariate and logistic regression analyses were performed to report on the potential risk factors associated with poor teeth and poor gum conditions of school children.

**Results:**

Psychometric analyses revealed that the Arabic Child Oral Health Assessment Tool (A-OHAT) was reliable and valid. A total of 478 (N) high school children were subjected to the tested tool, of which 66.5% were male and 33.5% were female with a mean age of 16.28 + 1.04 years. 80.3% of school children had poor teeth condition and 36.2% of school children had often experienced toothache. Children had 1.5 times higher odds of having poor teeth condition if they had increased frequency of sweet and candy consumption. It was also seen that increased frequency of sweets and candy consumption by school children had put them at nearly 20% higher risk of having poor gum condition. Finally, children with the habit of using toothbrush had nearly 50% lower chance of having poor gum condition in contrast to the school children who do not use toothbrush.

**Conclusion:**

To conclude, the study provides a reliable and valid tool to assess the oral health status of Arab adolescents. Improper oral hygiene habits and diet were identified as the plausible risk factors for poor teeth and gum condition.

## Background

Oral health is related to the overall health of individuals [1], and Petersen in the bulletin of World Health Organization (WHO) had also emphasized that, “good oral health is an integral part of general health” [2]. It is further informed that tooth decay and gum diseases are the two most common oral health problems affecting school children globally [2]. Children are the future of any nation and their health and wellbeing should be of utmost importance. These oral health problems if persisted among the children quite frequently may lead to pain, speech impairment, sleep disruption, difficulty in eating and growth disorders [3].

Epidemiologic studies report that the distribution of these oral diseases and the associated risk factors vary in different parts of the world and sometimes they also show variation within the same country or region [4]. A recent systematic review revealed that the prevalence of tooth decay is very high among Saudi Arabian school children in comparison to other developed and developing nations in the Eastern Mediterranean region [5]. A study conducted in the capital city of Saudi Arabia showed that 77.7% of school children aged 12–13 years had been suffering from dental caries [6]. Few years later, another study in the same city demonstrated a much increased prevalence rate (94.4%) of tooth decay [7]. Other less developed regions like Jazan, which borders Yemen have also exhibited similar data on oral health status of children and adolescents [8, 9].

Assessment of risk factors by Amin and Al Abad had unveiled that the increased exposure to sugared food and beverages was strongly related to the high risk of oral diseases among the school children of Riyadh [10]. Thus, there is a need to combat these oral health problems and data from diverse regions will contribute to the essential nationwide or region-based reports of these highly prevalent diseases.

The status of oral health and the associated risk factors can be assessed using multiple methods. But, most of them remain unstandardized and do not contribute to the uniformity in data representation across the nations. The World Health Organization (WHO) has provided a questionnaire for assessment of child oral health on their website, titled: “Oral health surveys: Basic methods – 5th Edition [11]. This assessment tool has a range of questions that not only evaluate the oral health status of the respondents but also identify the involvement of potential risk factors. Such tools are easy to administer even by undergraduate students, interns and dental auxiliaries in large populations. Some studies have also stated the use of these kind of tools by non-dental professionals in tertiary hospitals to gather data on the oral health status of their patients [12]. Currently, there is no Arabic version of the WHO oral health assessment tool for children. Availability of such tool will aid in collecting oral health data among child population using a unified questionnaire among the various Arabic speaking nations.

Thus, the objectives of the current study are: To validate the Arabic version of WHO child oral health assessment tool (A-OHAT); to assess the oral health status of high school children in Gizan region of Saudi Arabia; and finally, to identify the important risk factors associated with poor teeth and gum conditions of these school children. We hypothesize that the oral health status of Arab children with improper oral hygiene is poor in comparison to the children with good oral hygiene habits.

## Methods

### Study design and study sample

A cross-sectional study was designed and high school students residing in the Jazan region were targeted. In order to maintain the homogeneity of the study sample, only the Arab nationals studying in the public schools were included and the children from other nationalities were excluded. Adolescents were preferred as target population following the recommendation provided by the original version of the WHO oral health assessment tool [11]. This also accounted for clear interpretation of oral health status from a single set of dentitions instead of mixed dentition.

Two-staged simple random sampling technique was conducted to obtain a near-representative sample. In the first stage, random selection of four male and four female schools was done after obtaining the complete list of schools from the director’s office. In the second stage, all the male and female children from the selected schools were approached. All of these students were given a consent form along with a brief description of the study proposal in order to keep the parents informed about their child’s participation in this study. A minimum sample size required for this study was 400, with an absolute precision of 0.05, expected proportion of 0.5 and an estimated design effect of 1 [9].

### Reliability and validity statistics for A-OHAT

The principal investigator had performed the forward translation into Arabic due to his fluency in both Arabic and English languages. This was followed by backward translation by a bilingual expert who was not part of the current study. Both, forward as well as backward translations were performed by taking into consideration the literal meaning of words and also the conceptual understanding of each item (question). Minor discrepancies in the Arabic version were adjusted after mutual consensus of both the translators.

A group of fifty school children were randomly selected as respondents for the pilot study to perform the psychometric analysis of A-OHAT. The sample of 50 was suggested based on the recommendation provided by Connelly, 2008 wherein 10% of the actual sample was deemed appropriate for pilot testing [13]. Internal consistency of the translated questionnaire was explored using Chronbach’s alpha and the reliability was calculated using the Intra class correlation (ICC). A paired t-test was performed to identify the differences between the readings after repeated administration of the assessment tool with a gap of 2 weeks [14].

In order to make sure that the items in the translated tool were assessing the intended isolated constructs; a convergent validity test was conducted. Spearman’s correlation coefficient was performed by identifying the association between perceived oral health status of OHIP-14 (Oral Health Impact Profile) and A-OHAT [15]. It was hypothesized that both of these questionnaires were revealing similar self-reported oral health status. The attached appendices demonstrate the individual items for English (Additional file 1) as well as the Arabic (Additional file 2) version of the WHO assessment tool, respectively.

### Data collection procedure

Validated A-OHAT, a self-oral health assessment tool was administered to the children from the selected high schools after the signed consents from their parents were obtained. Sixth-year dental undergraduate students of Jazan University had assisted in distributing the questionnaire as part of their Community Dental Practice course (Course code – 632 PDS). Male undergraduate students approached the male high schools, while female undergraduate students approached the female high schools. On an average, the school children took twenty minutes to mark their responses.

### Statistical analysis

After receiving the completed A-OHAT from the respondents, the data was recorded and analyzed using the IBM - SPSS version 24. Missing values and normality distribution were checked prior to analyses. Gender variation of perceived oral health and the associated risk factors was checked using Chi-Square test. This was done intuitively, as the male and female school children are subjected to separate schooling environments. The responses obtained from Five-point Likert scale on teeth condition and the gum condition was dichotomized into “good / poor” teeth and gum conditions, respectively. Very good / Good and Average were collapsed to ‘Good condition’; whereas; Poor and Very poor were combined as ‘Poor Condition’, for both teeth and gum conditions, respectively. Finally, logistic regression was conducted separately to identify the potential risk factors associated with poor teeth condition and poor gum condition.

## Results

### Psychometric properties of A-OHAT

The mean intra class correlation (ICC) from the pilot sample of fifty school children to test the reliability on both the occasions with a gap of 2 weeks was 0.89 + 0.08; and experts suggest that an ICC value of more than 0.75 gives excellent reliability [16]. Test – retest reliability using paired t-test revealed no significant difference (*P* > 0.05). The Cronbach alpha value obtained was 0.72, which indicated a desirable internal consistency [17] (Table 1).

**Table 1:** Reliability statistics values for Arabic-WHO assessment tool

Instrument	ICC	Cronbach’s Alpha
A-WHO	0.89 + 0.08	0.72

*ICC* Intra class correlation

For identifying the convergent validity, all items were grouped under three broad constructs. Items 1, 2, 3, 16 and 17 described the demographic construct; items 4 and 5 described the oral health condition construct and finally; items 6, 7, 8, 9, 10, 11, 12, and 15 described the oral habits construct. Item correlational statistics indicated that each item under their respective constructs was significantly correlated (Table 2). Responses to the global construct of oral health condition after dichotomization into Good/Poor revealed significantly higher value for the latter. Spearman correlation coefficients value of *r* = 1.05, *P* < 0.05 was obtained between perceived oral health status of OHIP-14 (Oral health impact profile) and A-OHAT.

**Table 2:** Item correlational statistics of oral health condition and the oral habits constructs

Oral Health Condition Construct^a^										
	Item 4	Item 5								
Item 4	1.00									
Item 5	0.83	1.00								
Oral Habits Construct^a^										
			Item 6	Item 7	Item 8	Item 9	Item 10	Item 11	Item 12	Item 15
Item 6			1.00							
Item 7			0.89	1.00						
Item 8			0.91	0.86	1.00					
Item 9			0.83	0.90	0.85	1.00				
Item 10			0.83	0.91	0.90	0.89	1.00			
Item 11			0.85	0.89	0.89	0.83	0.90	1.00		
Item 12			0.90	0.86	0.85	0.90	0.86	0.83	1.00	
Item 15			0.86	0.80	0.90	0.86	0.85	0.90	0.86	1.00

^a^Item Confirmatory factor analysis

### Descriptive analysis of study sample

After the administration of the validated A-OHAT, the descriptive and regression analyses were performed. In this cross-sectional study, a total of 478 (N) high school children participated, out of which 66.5% were male and 33.5% were female. Mean age of the study sample was 16.28 + 1.04 years. Nearly, 42% of school children came from urbanized locations of Jazan region and 58% came from rural locations. Most of the school children had reported that their father (43%) and mother (36%) had completed the university level education, respectively (Table 3).

**Table 3:** Demographic characteristics of the study sample

Variable	Frequency	Percentage
Gender		
Male	318	66.5%
Female	160	33.5%
Location		
Urban	202	42.3%
Rural	276	57.7%
Fathers Education		
No formal schooling	15	3.1%
Less than primary school	10	2.1%
Primary school completed	24	5.0%
Secondary school completed	37	7.7%
High school completed	94	19.7%
University completed	205	42.9%
No adult male in household	7	1.5%
Mothers Education		
No formal schooling	47	9.8%
Less than primary school	8	1.7%
Primary school completed	43	9.0%
Secondary school completed	56	11.7%
High school completed	83	17.4%
University completed	173	36.2%
No adult female in household	3	0.6%

Mean age of the study population was 16.28 + 1.04

Considering the fact that the male and female school children in Saudi Arabia are segregated into different campus environments, the authors of this study first assessed the gender variation of the dependent variable (Oral health condition). This was necessary in order to provide a clear pathway for further regression analysis, but the results suggested no significant (*P* > 0.05) variation (Table 4). Hence, further analyses were combined for male and female school children.

**Table 4:** Gender variation of oral health problems among school children

Oral health Status	Gender		Total	*P-Value
	Boy *N* = 318	Girl *N* = 160		
Condition of teeth				
Excellent	4 (1.2%)	4 (2.5%)	8 (1.7%)	0.72
Very good	12 (3.7%)	6 (3.7%)	18 (3.8%)	
Good	38 (11.9%)	16 (10.0%)	54 (11.3%)	
Average	0	0	0	
Poor	257 (80.8%)	127 (79.3%)	384 (80.3%)	
Very poor	0	0	0	
Condition of gums				
Excellent	56 (17.6%)	26 (16.2%)	82 (17.2%)	0.39
Very good	107 (33.6%)	53 (33.1%)	160 (33.5%)	
Good	62 (19.4%)	26 (16.2%)	88 (18.4%)	
Average	51 (16.0%)	33 (20.6%)	84 (17.6%)	
Poor	21 (6.6%)	7 (4.3%)	28 (5.9%)	
Very poor	16 (5.0%)	14 (8.7%)	30 (6.3%)	
Toothache within past 6 months				
Often	117 (36.7%)	56 (35.0%)	173 (36.2%)	0.21
Occasionally	77 (24.2%)	30 (18.7%)	107 (22.4%)	
Rarely	67 (21.0%)	22 (13.7%)	89 (18.6%)	
Never	38 (11.9%)	25 (15.6%)	63 (13.2%)	
Difficulty of chewing in past 6 months				
No	18 (5.6%)	11 (6.8%)	29 (6.1%)	0.13
Yes	70 (22.0%)	21 (13.1%)	91 (19.0%)	
Don’t Remember	201 (63.2%)	100 (62.5%)	301 (63.0%)	
Difficulty to bite hard food				
No	45 (14.1%)	19 (11.8%)	64 (13.4%)	0.36
Yes	37 (11.6%)	12 (7.5%)	49 (10.3%)	
Don’t Remember	213 (66.9%)	110 (68.7%)	323 (67.6%)	
Avoid smile due poor oral health				
No	1 (0.3%)	0	1 (0.2%)	0.31
Yes	87 (27.3%)	56 (35.0%)	143 (29.9%)	
Don’t Remember	210 (66.0%)	102 (63.7%)	312 (65.3%)	
Friends make fun of my teeth				
No	32 (10.1%)	13 (8.1%)	45 (9.4%)	0.10
Yes	154 (48.4%)	63 (39.3%)	217 (45.4%)	
Don’t Remember	120 (37.7%)	75 (46.8%)	195 (40.8%)	
Dissatisfied with my teeth appearance				
No	13 (4.1%)	3 (1.8%)	16 (3.3%)	0.13
Yes	291 (91.5%)	157 (98.1%)	448 (93.7%)	

*P-value determined using chi-square analysis

Findings showed that 80.3% of school children had poor teeth condition and 36.2% of school children had often experienced toothache. It was also seen that nearly 93% of high school children were not satisfied with the appearance of their teeth (Table 4). Gender variation of independent variables revealed significant difference between the diet consumption and oral hygiene habits among male and female school children (Table 5). Higher frequency of sweet food and carbohydrate consumption was seen in male school children in contrast to female school children (Table 5).

**Table 5:** Description of independent variables among male and female school children

Diet	Gender		Total	*P*-Value
Fruits	Male *N* = 318	Female *N* = 160		
Never	19 (6%)	24 (15%)	43 (9.0%)	< 0.001*
Several times a month	61 (19.2%)	46 (28.7%)	107 (22.4%)	
Once a week	58 (18.2%)	26 (16.2%)	84 (17.6%)	
Several times a week	94 (29.5%)	37 (23.1%)	131 (27.4%)	
Everyday	55 (17.3%)	12 (7.5%)	67 (14.0%)	
Several times a day	29 (9.1%)	14 (10.6%)	43 (9.0%)	
Lemonade				
Never	17 (5.3%)	11 (6.9%)	28 (5.9%)	0.50
Several times a month	20 (6.3%)	15 (9.4%)	35 (7.3%)	
Once a week	20 (6.3%)	13 (8.1%)	33 (6.9%)	
Several times a week	57 (17.9%)	31 (19.4%)	88 (18.4%)	
Everyday	123 (38.7%)	50 (31.2%)	173 (36.9%)	
Several times a day	77 (24.2%)	35 (21.9%)	112 (23.9%)	
Jam and Honey				
Never	85 (26.7%)	70 (43.7%)	155 (32.4%)	< 0.001*
Several times a month	69 (21.7%)	35 (21.8%)	104 (21.8%)	
Once a week	39 (12.3%)	21 (13.1%)	60 (12.6%)	
Several times a week	66 (20.7%)	20 (12.5%)	86 (18.0%)	
Everyday	34 (10.7%)	3 (1.9%)	37 (7.7%)	
Several times a day	14 (4.4%)	6 (3.7%)	20 (4.2%)	
Sweet and Candy				
Never	27 (8.5%)	12 (7.5%)	39 (8.2%)	0.63
Several times a month	22 (6.9%)	15 (9.4%)	37 (7.7%)	
Once a week	30 (9.4%)	12 (7.5%)	42 (8.8%)	
Several times a week	84 (26.4%)	36 (22.5%)	120 (25.1%)	
Everyday	96 (30.2%)	48 (3%)	144 (30.1%)	
Several times a day	54 (17%)	35 (21.9%)	89 (18.6%)	
Milk with Sugar				
Never	24 (7.5%)	8 (5%)	32 (6.7%)	< 0.001*
Several times a month	25 (7.9%)	10 (6.2%)	35 (7.3%)	
Once a week	44 (13.8%)	13 (8.1%)	57 (11.9%)	
Several times a week	94 (29.5%)	26 (16.2%)	120 (25.1%)	
Everyday	78 (24.5%)	59 (36.9%)	137 (28.7%)	
Several times a day	45 (14.5%)	42 (26.2%)	87 (18.2%)	
Tea with Sugar				
Never	69 (21.7%)	41 (25.6%)	110 (23%)	0.002*
Several times a month	46 (14.5%)	36 (22.5%)	82 (17.2%)	
Once a week	42 (13.2%)	20 (12.5%)	62 (13.0%)	
Several times a week	73 (23%)	27 (16.9%)	100 (20.9%)	
Everyday	61 (19.2%)	13 (8.1%)	74 (15.5%)	
Several times a day	22 (7%)	20 (12.5%)	42 (8.8%)	
Coffee with Sugar				
Never	40 (12.6%)	30 (18.7%)	70 (14.6%)	0.05*
Several times a month	31 (9.7%)	23 (14.3%)	54 (11.3%)	
Once a week	37 (11.6%)	26 (16.2%)	63 (13.2%)	
Several times a week	85 (26.7%)	33 (20.6%)	118 (24.7%)	
Everyday	76 (23.9%)	26 (16.2%)	102 (21.3%)	
Several times a day	42 (13.2%)	20 (12.5%)	62 (13.0%)	

*Significant *P*-value determined using chi-square analysis

### Multivariate analysis of study sample

Logistic regression was performed to identify the independent variables that contributed to the poor oral health of school children. Factors affecting teeth condition (Table 6) and gum condition (Table 7) were identified independently after adjusting for gender, location, and parent’s education. It was seen that frequency of consumption of sweet and candy was significantly (*P* = 0.04) related to poor teeth condition. The school children had 1.5 times higher odds of having poor teeth condition if they had increased frequency of sweet and candy consumption (Table 6). While the assessment of factors impacting the gum-condition showed that frequency of teeth cleaning, frequency of routine dental visits, use of toothbrush and consumption of sweets and candy were significantly (*P* < 0.05) associated (Table 7). More specifically, increased frequency of sweets and candy consumption by school children had put them at nearly 3 times higher odds of having poor gum condition (Table 7). Also, school children who have the habit of using toothbrush had nearly 50% lower chance of having poor gum condition in contrast to the school children who do not use toothbrush (Table 7).

**Table 6:** Risk factors affecting teeth condition of school children

Risk factors	Teeth Condition		*P*-Value	Adjusted ^a^ O.R	Confidence Interval	
	Good	Poor			Minimum	Maximum
Teeth Cleaning Frequency						
Once	3	18	0.26	0.91	0.77	1.07
Twice	7	32				
Three times	7	17				
Four times	9	37				
More than four times	27	129				
Routine Dental Visit						
Once	64	16	0.12	0.84	0.67	1.04
Twice	87	30				
Three Times	142	19				
Four Times	69	7				
More than 4 times	33	8				
Sweets and Candy						
Never	33	6	0.04*	1.50	1.00	1.44
Several times a month	33	4				
Once a week	35	7				
Several times a week	8	112				
Everyday	35	109				
Several times a day	20	69				
Fluoridated Toothpaste						
Yes	2	12	0.98	0.98	0.20	4.65
No	78	386				
Mothers Education						
No Schooling	10	37	0.34	0.94	0.82	1.06
Less than primary school	3	5				
Primary School Completed	5	38				
Secondary School Completed	15	41				
High School Completed	23	60				
University Completed	14	159				

*Significant *P*-value determined using chi-square analysis

*O.R* Odds ratio determined using ENTER method

^a^ *O. R* Adjusted for gender, location and parent’s education

**Table 7:** Risk factors affecting gum condition of school children

Risk factorsa	Gum Condition		*P*-Value	Adjusted ^a^ O.R	Confidence Interval	
	Good	Poor			Minimum	Maximum
Teeth Cleaning Frequency	(*N* = 190)	(*N* = 59)				
Once	10 (52%)	11 (18.6%)	0.02*	1.18	1.02	1.36
Twice	25 (13.1%)	14 (23.7%)				
Three times	15 (7.8%)	9 (15.2%)				
Four times	32 (16.8%)	14 (23.7%)				
More than four times	108 (56.8%)	11 (18.6%)				
Routine Dental Visit	(*N* = 330)	(*N* = 145)				
Once	47 (14.2%)	33 (22.7%)	< 0.001*	1.39	1.15	1.68
Twice	61 (18.4%)	56 (38.6%)				
Three Times	130 (39.3%)	31 (21.3%)				
Four Times	62 (18.7%)	14 (9.6%)				
More than 4 times	30 (9.0%)	11 (75%)				
Sweets and Candy	(*N* = 160)	(*N* = 311)				
Never	27 (16.8%)	12 (3.8%)	0.05*	2.86	0.74	3.00
Several times a month	11 (6.8%)	26 (8.3%)				
Once a week	8 (5%)	34 (10.9%)				
Several times a week	29	91				
Everyday	51 (31.8%)	93 (29.9%)				
Several times a day	34 (21.2%)	55 (17.6%)				
Toothbrush	(*N* = 330)	(*N* = 148)				
Yes	300 (90.9%)	124 (83.7%)	0.04*	0.49	0.25	0.96
No	30 (9%)	24 (16.2%)				
Smokeless Tobacco	(*N* = 139)	(*N* = 317)				
Never	135 (97.1%)	298 (94%)	0.15	1.31	0.90	1.92
Seldom	2 (1.4%)	8 (2.5%)				
Several Times In Month	0	1 (0.3%)				
Several Times A Week	1 (0.7%)	2 (0.6%)				
Every Day	1 (0.7%)	8 (2.5%)				
Smoked Tobacco	(*N* = 308)	(*N* = 137)				
Never	277 (89.9%)	124 (90.5%)	0.95	1.00	0.83	1.21
Seldom	16 (5.1%)	4 (2.9%)				
Several Times In Month	8 (2.5%)	3 (2.1%)				
Several Times A Week	3 (0.9%)	2 (1.4%)				
Every Day	4 (1.2%)	4 (2.9%)				

*Significant *P*-value determined using chi-square analysis

*O.R* Odds ratio determined using ENTER method

^a^ *O. R* Adjusted for gender, location and parent’s education

## Discussion

The current study is first to provide a WHO oral health self-assessment tool (A-OHAT) that can be administered to a wide range of Arabic speaking child populations, especially in the Eastern Mediterranean region (Additional file 2). Findings of psychometric analyses from the current study are in accordance with a similar WHO oral health assessment tool constructed for adults and tested for its reliability and validity [18]. The current study is also the first to describe the oral health status of high school children (Mean age = 16 years) residing in Jazan region of Saudi Arabia utilizing a self-assessment tool. The high prevalence of school children with poor teeth condition observed in the current study was similar to the reports from other developing [19] as well as developed nations [20] among children of same age range. However, there are no results from similar age group in Jazan region for comparison, but studies conducted in much younger children have also shown high prevalence of clinically examined tooth decay [8, 9, 21].

Further, results from the current study had revealed that both male and female children have quite often experienced toothache, and this may subsequently affect their daily activities [22] and also increase the possibility of school absenteeism [23]. These types of associations through the application of Oral health related Quality of Life assessment tools like Child-Oral Impact on Daily Performances (Child-OIDP) have to be further explored in the Arab population.

The assessment of potential risk factors indicated that the increased frequency of sweet food consumption among the adolescents was significantly associated with their poor teeth condition and this finding was in accordance with an earlier study performed among children of different age groups in this region [9]. The gum condition of the study population was also reported to be poor by most of the school children. Potential risk factors related with poor gum condition were identified as: ‘fewer visits to oral health care providers’, decreased ‘frequency of mouth cleaning’ and ‘not using the toothbrush’. Studies conducted elsewhere have also put forth that poor oral hygiene maintenance is directly related with poor gum condition [24–26]. It is to be noted that the dental care in Saudi Arabia is free and managed by the Ministry of Health (MoH) with nearly 314 tertiary hospital and 1756 primary health centers. Each governorate has an access to at least one primary dental center which acts as a referral route to more equipped dental hospitals [10]. Ironically, the utilization rates of oral health care among the children and adolescents which could act as a potential risk factor for oral health status of the population has not been studied and reported [27].

In addition to the above mentioned factors, some of the previous reports have also mentioned that ‘mothers education’ and ‘family income’ are closely associated with gingival inflammation [25]. On the contrary, these factors have not shown any influence on poor gum condition in the current sample. This could be attributed to the homogeneity maintained among the study population. On searching the literature, it is observed that there are very few published studies from the middle-east region which report on the gum condition of school children and its associated risk factors. The Global data provided by WHO have stated that more than 80% of the child population across the world suffers from some or the other form of gum problems [11]. This suggests that there is a need of further studies to explore the grade of gum inflammation and also the associated risk factors in this region.

Robust psychometric analysis and random selection of the participants is the strength of the study and it demonstrates the internal and external validity of the study. During the initial forward and backward translation, the Arabic-WHO child oral health assessment tool reflected the quality and originality of its English version [11]. The tested A-OHAT is extremely easy to administer and the authors recommend that this tool could be utilized by health auxiliaries, graduating students and in many cases by school authorities (Educators) to identify oral health status of their school children. Thus, contributing to the early detection of oral health problems in children so that appropriate and timely measures are initiated. Further, we also suggest that such assessment tools can be applied in multi-center studies were data from different regional populations could be gathered in a much easier fashion maintaining the generalizability. The data gathered will thus contribute to global findings through a standardized assessment tool.

However, the current study has its limitations that are important to be considered during the interpretation of the findings. The administrative tool determines the oral health status subjectively and clinical examination of oral cavity will provide more accurate and comprehensive findings. Also, the design is cross-sectional and hence does not demonstrate the temporality between the oral health conditions and the potential risk factors.

## Conclusions

To conclude, the current study provides a reliable and valid tool to assess the oral health status of children in Arabic speaking nations. The teeth and gum condition of the Arab adolescents in this study was generally poor; and improper oral hygiene habits and diet were identified as the plausible risk factors.

## Supplementary information

**Additional file 1.** WHO-OHAT Demonstrates the individual items for the English version of the Oral Health Assessment Tool provided by the World Health Organization.

**Additional file 2.** A-OHAT Demonstrates the individual items for the current validated Arabic version of the Oral Health Assessment Tool.

## Data Availability

The data used to support the findings of this study are available from the corresponding author upon request.

## Abbreviations

A-OHAT: Arabic-Oral health assessment tool
ICC: Intra class correlation
OHIP: Oral health impact profile
OIDP: Oral impact on daily performance
WHO: World Health Organization
