# Supplementary material for: What factors contribute to the self-reported oral health status of Arab adolescents? An assessment using a validated Arabic-WHO tool for child oral health (A-OHAT)
Source: BMC Oral Health. 2020 Jan 28;20:21. doi: 10.1186/s12903-020-1018-8 (PMC6986000; doi:10.1186/s12903-020-1018-8)
Supplement: Supplementary file 1 — Additional file 1. WHO-OHAT Demonstrates the individual items for the English version of the Oral Health Assessment Tool provided by the World Health Organization. [file 12903_2020_1018_MOESM1_ESM.pdf]

World Health Organization

Annex 8

Oral Health Questionnaire for Children

First, we would like you to answer some questions concerning yourself and your teeth

	**Identification number**	**Sex**		**Location**		
		Boy	Girl	Urban	Periurban	Rural
1.	☐☐☐☐	☐	☐	☐	☐	☐
	1 4	1	2	1	2	3

2. **How old are you today?** ____________________
(Years)

3. **How would you describe the health of your teeth and gums?**

(Read each item)

	**Teeth**	**Gums**
Excellent	☐ 1	☐ 1
Very good	☐ 2	☐ 2
Good	☐ 3	☐ 3
Average	☐ 4	☐ 4
Poor	☐ 5	☐ 5
Very poor	☐ 6	☐ 6
Don't know	☐ 9	☐ 9

4. **How often during the past 12 months did you have toothache or feel discomfort due to your teeth?**

Often	☐ 1
Occasionally	☐ 2
Rarely	☐ 3
Never	☐ 4
Don't know	☐ 9

Now please answer some questions about the care of your teeth

5. **How often did you go to the dentist during the past 12 months?**
(Put a tick/cross in one only)

Once .... ☐ 1
Twice .... ☐ 2
Three times .... ☐ 3
Four times .... ☐ 4
More than four times .... ☐ 5
I had no visit to dentist during the past 12 months .... ☐ 6
I have never received dental care/visited a dentist .... ☐ 7
I don’t know/don’t remember .... ☐ 9

If you did not see a dentist during the last 12 months, go on to question 7

6. **What was the reason for your last visit to the dentist?**
(Put a tick/cross in one box only)

Pain or trouble with teeth, gums or mouth .... ☐ 1
Treatment/follow-up treatment .... ☐ 2
Routine check-up of teeth/treatment .... ☐ 3
I don’t know/don’t remember .... ☐ 9

7. **How often do you clean your teeth?**
(Put a tick/cross in one box only)

Never .... ☐ 1
Several times a month (2–3 times) .... ☐ 2
Once a week .... ☐ 3
Several times a week (2–6 times) .... ☐ 4
Once a day .... ☐ 5
2 or more times a day .... ☐ 6

8. **Do you use any of the following to clean your teeth or gums?**
(Read each item)

	Yes 1	No 2
Toothbrush	☐	☐
Wooden toothpicks	☐	☐
Plastic toothpicks	☐	☐
Thread (dental floss)	☐	☐
Charcoal	☐	☐
Chewstick/miswak	☐	☐
Other	☐	☐

Please specify______________________________

9.

	Yes	No
a) **Do you use toothpaste to clean your teeth**	☐ 1	☐ 2

	Yes	No
b) **Do you use toothpaste that contains fluoride?**	☐ 1	☐ 2

Don't know....... ☐ 9

10. **Because of the state of your teeth and mouth, have you experienced any of the following problems during the past year?**

	Yes 1	No 2	Don't know 0
(a) I am not satisfied with the appearance of my teeth	☐	☐	☐
(b) I often avoid smiling and laughing because of my teeth	☐	☐	☐
(c) Other children make fun of my teeth	☐	☐	☐
(d) Toothache or discomfort caused by my teeth forced me to miss classes at school or miss school for whole days	☐	☐	☐
(e) I have difficulty biting hard foods	☐	☐	☐
(f) I have difficulty in chewing	☐	☐	☐

11. **How often do you eat or drink any of the following foods, even in small quantities?**
(Read each item)

	Several times a day	Every day	Several times a week	Once a week	Several times a month	Never
	6	5	4	3	2	1
Fresh fruit	☐	☐	☐	☐	☐	☐
Biscuits, cakes, cream cakes, sweet pies, buns etc.	☐	☐	☐	☐	☐	☐
Lemonade, Coca Cola or other soft drinks	☐	☐	☐	☐	☐	☐
Jam/honey	☐	☐	☐	☐	☐	☐
Chewing gum containing sugar	☐	☐	☐	☐	☐	☐
Sweets/candy	☐	☐	☐	☐	☐	☐
Milk with sugar	☐	☐	☐	☐	☐	☐
Tea with sugar	☐	☐	☐	☐	☐	☐
Coffee with sugar	☐	☐	☐	☐	☐	☐

(Insert country-specific items)

12. **How often do you use any of the following types of tobacco?**
(Read each item)

	Every day	Several times a week	Once a week	Several times a month	Seldom	Never
	6	5	4	3	2	1
Cigarettes, pipe or cigars	☐	☐	☐	☐	☐	☐
Chewing tobacco or snuff	☐	☐	☐	☐	☐	☐

13. **What level of education has your father completed (or your stepfather, guardian or other male adult living with you)?**

- No formal schooling...☐ 1
- Less than primary school ....☐ 2
- Primary school completed...☐ 3
- Secondary school completed...☐ 4
- High school completed ....☐ 5
- College/university completed ....☐ 6
- No male adult in household ....☐ 7
- Don't know ....☐ 9

14. **What level of education has your mother completed?**

- No formal schooling...☐ 1
- Less than primary school ....☐ 2
- Primary school completed...☐ 3
- Secondary school completed...☐ 4
- High school completed ....☐ 5
- College/university completed ....☐ 6
- No female adult in household ....☐ 7
- Don't know ....☐ 9

(Insert country-specific categories)

That completes our questionnaire

Thank you very much for your cooperation!

Year	Month	Day	Interviewer	District	Country
☐☐	☐☐	☐☐	☐☐	☐☐	☐☐☐☐
