# Supplementary material for: What factors contribute to the self-reported oral health status of Arab adolescents? An assessment using a validated Arabic-WHO tool for child oral health (A-OHAT)
Source: BMC Oral Health. 2020 Jan 28;20:21. doi: 10.1186/s12903-020-1018-8 (PMC6986000; doi:10.1186/s12903-020-1018-8)
Supplement: Supplementary file 2 — Additional file 2. A-OHAT Demonstrates the individual items for the current validated Arabic version of the Oral Health Assessment Tool. [file 12903_2020_1018_MOESM2_ESM.pdf]

2019

استبيان منظمة الصحة العالمية (WHO) لمعاينة صحة الفم والأسنان

كلية طب الأسنان - جامعة جازان

اختر الإجابة الصحيحة التي تعبر عن نفسك وعن حالة أسنانك الصحية:

1) **مدينة السكن الحالية: ____________________**

2) **الجنس** 1 ☐ **ذكر** 2 ☐ **أنثى**

3) **كم عمرك اليوم؟** ☐ **شهر** ☐ **سنة**

4) **كم عدد الأسنان الطبيعية التي تمتلكها؟**

**لا توجد لدي أي أسنان طبيعية ....**	1 ☐
**1- 9 أسنان ....**	2 ☐
**10-19 سن ....**	3 ☐
**20 سن أو أكثر ....**	4 ☐

5) **كيف تصف الحالة الصحية لأسنانك ولثتك؟**

	الأسنان	اللثة
**ممتازة ....**	1 ☐	1 ☐
**جيدة جداً ....**	2 ☐	2 ☐
**جيدة ....**	3 ☐	3 ☐
**متوسطة ....**	4 ☐	4 ☐
**سيئة ....**	5 ☐	5 ☐
**سيئة جداً ....**	6 ☐	6 ☐
**لا أعلم ....**	7 ☐	7 ☐

6) خلال ال١٢ شهراً، هل حدث وأن آلمتك أسنانك أو شعرت بشيء من عدم الراحة بسببها؟
(اقرأ كل الاختيارات)

غالباً .... 1 ☐

من حين إلى آخر .... 2 ☐

نادراً .... 3 ☐

أبداً لم يحدث .... 4 ☐

لا أعلم .... 5 ☐

الآن رجاءً أجب على بعض الأسئلة التي تتعلق بصحة أسنانك

7) كم مرة ذهبت إلى طبيب الأسنان خلال ال١٢ شهراً الماضية؟
(اختر إجابة واحدة/ ضع علامة X على الإجابة الصحيحة)

مرة واحدة .... 1 ☐

مرتان .... 2 ☐

ثلاثة مرات .... 3 ☐

أربعة مرات .... 4 ☐

أكثر من أربعة مرات .... 5 ☐

لم أذهب ولا مرة لزيارة طبيب الأسنان خلال ال١٢ شهراً الماضية .... 6 ☐

لا أعلم / لا أتذكر .... 7 ☐

إذا لم تذهب إلى طبيب الأسنان بتاتاً خلال ال١٢ شهراً الماضية، اذهب إلى السؤال رقم (9)

8) ما السبب في زيارتك الأخيرة لطبيب الأسنان؟
(اختر إجابة واحدة/ ضع علامة X على الإجابة الصحيحة)

ألم أو مشكلة في الأسنان، اللثة أو الفم .... 1 ☐

علاج أو مراجعة ما بعد العلاج .... 2 ☐

كشف عام شامل .... 3 ☐

لا أعلم / لا أتذكر .... 4 ☐

9) كم مرة تنظف فيها أسنانك عادة؟
(اختر إجابة واحدة/ ضع علامة X على الإجابة الصحيحة)

أبداً .... 1 ☐

بعض الأحيان شهرياً (2-3 مرات) .... 2 ☐

مرة في الأسبوع .... 3 ☐

عدة مرات أسبوعياً (2-6 مرات) .... 4 ☐

مرة واحدة في اليوم .... 5 ☐

مرتان أو أكثر يومياً .... 6 ☐

١٠) هل تستخدم أياً من المواد التالية لتنظيف أسنانك أو لثتك؟
(اختر إجابة أو أكثر حسب ما يناسب حالتك)

فرشاة الأسنان .... 1 ☐

عود (خلال او نكاش) الأسنان الخشبي .... 2 ☐

عود (خلال او نكاش) الأسنان البلاستيك .... 3 ☐

خيط الأسنان .... 4 ☐

الفحم .... 5 ☐

المسواك .... 6 ☐

أخرى .... 7 ☐

الرجاء التوضيح: ______________________

١١) هل تستخدم معجون الأسنان عند تنظيف أسنانك؟

نعم .... 1 ☐

لا .... 2 ☐

١٢) هل تستخدم معجون أسنان يحتوي على مادة الفلورايد؟

نعم .... 1 ☐

لا .... 2 ☐

لا أعلم .... 3 ☐

13) بسبب حالة أسنانك وفمك الصحية، هل صادفتك أي مشكلة من المشاكل التالية خلال السنة الماضية؟

	نعم (1)	لا (2)	لا أعلم (0)
a) لم أكن راضياً عن المظهر الخارجي لأسناني	☐	☐	☐
b) غالباً أتجنب الابتسام والضحك بسبب مظهر أسناني	☐	☐	☐
c) الآخرون يسخرون من مظهر أسناني	☐	☐	☐
d) يوجد ألم أو عدم ارتياح بسبب أسناني مما أجبرني على التغيب عن الحصص أو المدرسة ليوم كامل	☐	☐	☐
e) أجد صعوبة في العض على الأكل القاسي	☐	☐	☐
f) أجد صعوبة في المضغ	☐	☐	☐

4 1) كم عادة تأكل أو تشرب أياً من المأكولات أو المشروبات التالية، وإن كانت بكميات قليلة؟

	عدة مرات في اليوم (6)	كل يوم (5)	عدة مرات في الأسبوع (4)	مرة في الأسبوع (3)	عدة مرات في الشهر (2)	أبداً (1)
a) فواكه طازجة	☐	☐	☐	☐	☐	☐
b) بسكويت، كعك، فطائر محلاه	☐	☐	☐	☐	☐	☐
c) عصيرات، مشروبات غازية	☐	☐	☐	☐	☐	☐
d) عسل أو مربى	☐	☐	☐	☐	☐	☐
e) علكة تحتوي على السكر	☐	☐	☐	☐	☐	☐
f) حلويات وسكاكر	☐	☐	☐	☐	☐	☐
g) حليب محلى بالسكر	☐	☐	☐	☐	☐	☐
h) شاي محلى بالسكر	☐	☐	☐	☐	☐	☐
i) قهوة محلاة بالسكر	☐	☐	☐	☐	☐	☐

15) كم مرة عادة تستخدم أياً من أنواع التبغ التالية؟

	كل يوم (6)	عدة مرات في الأسبوع (5)	مرة في الأسبوع (4)	عدة مرات في الشهر (3)	نادراً ما (2)	إطلاقاً (1)
السجائر، الشيشة/المعسل، السيجار	☐	☐	☐	☐	☐	☐
التبغ المضغي مثل: الشمة، التنبول	☐	☐	☐	☐	☐	☐

16) ما هو المستوى العلمي الذي حصل عليه والدك (في حال عدم وجوده: الوصي الشرعي مثل العم أو الخال أو زوج الأم)؟

لا تعليم رسمي .... 1 ☐

أقل من المرحلة الابتدائية .... 2 ☐

المرحلة الابتدائية .... 3 ☐

المرحلة المتوسطة .... 4 ☐

المرحلة الثانوية .... 5 ☐

المرحلة الجامعية/كلية .... 6 ☐

لا يوجد ولي أمر (ذكر) بالمنزل .... 7 ☐

لا أعلم .... 8 ☐

7 1) ما هو المستوى العلمي الذي حصلت عليه والدتك (الأم)؟

لا تعليم رسمي .... 1 ☐

أقل من المرحلة الابتدائية .... 2 ☐

المرحلة الابتدائية .... 3 ☐

المرحلة المتوسطة .... 4 ☐

المرحلة الثانوية .... 5 ☐

المرحلة الجامعية/كلية .... 6 ☐

لا توجد أنثى مربية بالمنزل .... 7 ☐

لا أعلم .... 8 ☐

انتهت الأسئلة ... شكراً لتعاونكم
